# Radioligand therapies in meningioma: Evidence and future directions

**DOI:** 10.1093/neuonc/noae069

**Published:** 2024-05-04

**Authors:** Maximilian J Mair, Emeline Tabouret, Derek R Johnson, Erik P Sulman, Patrick Y Wen, Matthias Preusser, Nathalie L Albert

**Affiliations:** Department of Nuclear Medicine, LMU Hospital, LMU Munich, Munich, Germany; Division of Oncology, Department of Medicine I, Medical University of Vienna, Vienna, Austria; Aix-Marseille Univ, APHM, CNRS, INP, Inst Neurophysiopathol, GlioME Team, plateforme PETRA, CHU Timone, Service de Neurooncologie, Marseille, France; Department of Radiology, Mayo Clinic, Rochester, Minnesota, USA; Brain and Spine Tumor Center, Laura and Isaac Perlmutter Cancer Center, NYU Langone, New York, New York, USA; Department of Radiation Oncology, New York University Grossman School of Medicine, New York, New York, USA; Center for Neuro-Oncology, Dana-Farber Cancer Institute, Boston, Massachusetts, USA; Department of Neurology, Brigham and Women’s Hospital and Harvard Medical School, Boston, Massachusetts, USA; Division of Oncology, Department of Medicine I, Medical University of Vienna, Vienna, Austria; Department of Nuclear Medicine, LMU Hospital, LMU Munich, Munich, Germany

**Keywords:** meningioma, nuclear medicine, positron emission tomography, radionuclide treatment, theranostic

## Abstract

Meningiomas are the most common intracranial neoplasms in adults. While most meningiomas are cured by resection, further treatment by radiotherapy may be needed, particularly in WHO grades 2 and 3 tumors which have an increased risk of recurrence, even after conventional therapies. Still, there is an urgent need for novel therapeutic strategies after the exhaustion of local treatment approaches. Radionuclide therapies combine the specificity of tumor-specific antibodies or ligands with the cytotoxic activity of radioactive emitters. Alongside this, integrated molecular imaging allows for a noninvasive assessment of predictive biomarkers as treatment targets. Whereas the concept of “theranostics” has initially evolved in extracranial tumors such as thyroid diseases, neuroendocrine tumors, and prostate cancer, data from retrospective case series and early phase trials underscore the potential of this strategy in meningioma. This review aims to explore the available evidence of radionuclide treatments and ongoing clinical trial initiatives in meningioma. Moreover, we discuss optimal clinical trial design and future perspectives in the field, including compound- and host-specific determinants of the efficacy of “theranostic” treatment approaches.

**Key Points**

1. Radionuclide treatments are approved in extracranial malignancies such as neuroendocrine tumors and metastatic prostate cancer based on pivotal clinical trial results.
2. Small, early-phase studies of somatostatin receptor (SSTR)-targeted radionuclide treatments suggest promising activity in meningioma. Well-designed clinical trials are needed to corroborate these findings.
3. Studies on the activity of different radionuclides as well as different routes of administration will deliver further insights guiding the development of future compounds.

Meningiomas are the most common intracranial neoplasms.^1^ Deriving from arachnoidal cap cells, the vast majority of meningiomas exhibit benign biological behavior, although some tumors show atypical features, invasive growth patterns and may even metastasize. This highly variable biological behavior is reflected in tumor grading according to the WHO Classification of Central Nervous System Tumors (current version 2021),^2^ classifying meningiomas into CNS WHO grades 1 (> 80% of all tumors), 2 (atypical, ~5%–20%) and 3 (anaplastic, ~2%).^3,4^ Whereas tumor grading was historically based on histological features alone, molecular factors are also increasingly considered. For instance, meningeal tumors with *CDKN2A/B* homozygous deletion are automatically defined as CNS WHO grade 3, and other molecular aberrations with prognostic impact have been described that may be included in future classification frameworks.^5^ Moreover, DNA methylation and gene expression profiling have resulted in an even more refined prognostic stratification.^6,7^

According to current guidelines, meningiomas can be cured by resection, and in asymptomatic patients even a watch-and-wait approach is justifiable.^8^ In cases where a gross total resection cannot be achieved and/or in higher-grade lesions, adjuvant radiotherapy or stereotactic radiosurgery may be necessary. Systemic treatment options may be used after exhaustion of local therapies, but are considered experimental as the underlying evidence is overall weak.^9^ New therapeutic options for salvage treatment are therefore urgently needed. Patient groups that could especially benefit from effective systemic therapy include those with tumors in locations that limit complete surgical resection (eg, skull base), patients with atypical and anaplastic meningioma due to higher recurrence rates, those with substantial symptomatic burden, and even rare cases of extracranial metastasis resulting in poor prognosis.

The concept of “theranostics” combines a “therapy” that is targeted at a “diagnostic” biomarker assessed by molecular imaging modalities. Initially, this approach was used in imaging studies and the therapy of both benign and well-differentiated malignant diseases of the thyroid gland using radioactive [^131^I]-iodine.^10^ More recently, additional compounds including targeted molecules linked to radionuclides have been developed and evaluated for clinical use in prostate cancer and neuroendocrine tumors (NETs), and further trials in other malignant diseases are ongoing.^11^ In this review, we aim to explore and discuss available data and future outlooks on “theranostics” approaches and radionuclide therapies in meningioma.

## Molecular Imaging of Meningiomas

One advantage of “theranostics” treatment strategies is the availability of noninvasive predictive biomarkers based on advanced imaging modalities. In standard imaging procedures such as computed tomography and magnetic resonance imaging (MRI), meningiomas classically present as contrast-enhancing extra-axial masses with a characteristic “dural tail.”^12^ Generally, these features allow for delineation of meningeal neoplasms with high certainty. However, challenging situations such as anatomical location in the skull base, atypical morphology such as “en plaque”-like growth patterns, distinction of vital tumor tissue from post-therapeutic changes in suspected recurrence, detection of residual tumor after surgery, and response assessment after radiotherapy or systemic treatment complicate the interpretation of meningioma imaging.

To overcome these challenges, molecular imaging techniques relying on the metabolic activity of neoplastic cells are increasingly considered in clinical routine. This includes [^18^F]fluoro-desoxy-glucose ([^18^F]FDG) positron emission tomography (PET) for the visualization of various extracranial tumors as well as the use of radiolabeled amino acids such as [^18^F]fluoroethyltyrosine (FET) or l-[*methyl*-^11^C]methionine in gliomas.^13^ In addition, radiolabeled ligands to specific receptors can be employed as PET tracers. Overall, 60%–100% of meningiomas express the somatostatin receptor type 2 (SSTR-2),^14–19^ and treatment with somatostatin analogs such as octreotide or pasireotide has been evaluated in small clinical trials.^20–22^ Tracers targeting SSTR are frequently used in nuclear imaging of meningeal neoplasms and generally show an improved tumor-to-background ratio compared to [^18^F]FDG and amino acid PET with almost no uptake in adjacent non-tumoral tissue such as brain and bone.^23,24^ Here, ^68^Ga-labeled tracers based on chemical modifications of the somatostatin receptor agonist octreotide are most frequently used, although ^64^Cu-labeled compounds are also applied. These include the widely available tracers [^68^Ga]gallium-DOTA-Tyr3-octreotide ([^68^Ga]Ga-DOTATOC), [^68^Ga]gallium-DOTA-Tyr3-octreotate ([^68^Ga]Ga-DOTATATE), and [^68^Ga]gallium-DOTA-1-NaI(3)-octreotide ([^68^Ga]Ga-DOTANOC).

Specific guidelines for SSTR-targeted PET imaging of meningiomas have jointly been elaborated by the European Association of Nuclear Medicine, the European Association of Neuro-Oncology, the Response Assessment in Neuro-Oncology working group (RANO), and the Society of Nuclear Medicine & Molecular Imaging (SNMMI).^25^ Comparative studies between used tracers are only available for NETs, where more lesions could be detected using [^68^Ga]Ga-DOTATOC and [^68^Ga]Ga-DOTANOC compared to [^68^Ga]Ga-DOTATATE.^26,27^ Conversely, a study in meningioma xenografts in mice showed higher uptake with [^68^Ga]Ga-DOTATATE compared to [^68^Ga]Ga-DOTATOC and [^68^Ga]Ga-DOTANOC.^28^ However, the clinical relevance of these subtle differences is limited given the already exceptional tumor-to-background ratio in meningiomas. Further tracers such as [^18^F]F-SiTATE based on a silicon fluoride acceptor (SiFA) are currently under investigation and may yield higher resolution and logistic advantages, given the longer half-life of ^18^F compared to ^68^Ga which results in lower radiation exposure and the possibility to centralize the production of larger lots of tracers.^29^

Prior to the advent of somatostatin receptor PET imaging, single photon emission computed tomography (SPECT) employing SSTR-2-targeted tracers such as [^111^In]In-pentetreotide or [^99^Tc]Tc -EDDA/HYNIC-TOC was widely used,^30,31^ but use today is mostly limited to circumstances where PET scanners are not available. Indeed, advantages of PET over SPECT imaging include higher spatial resolution, improved sensitivity, lower radiation dose to the patient, shorter exam duration, and greater capacity for quantitative information.^32^

For clinical applications, PET imaging of meningiomas is recommended in situations where the extent of the tumor or the distinction of recurrence from treatment-related changes is unclear,^8^ and SSTR-directed PET may be particularly helpful in the presurgical planning of meningiomas with intraosseus spread.^33^ In addition, postoperative PET might support the estimation of the extent of resection, as detection rates were improved compared to postoperative MRI.^34,35^

## Evidence for Radionuclide Treatment Efficacy in Extracranial Solid Tumors

In the 1930s, increasing knowledge of the effects of radioactivity on human tissue and iodine physiology of the thyroid gland provided the basis for radioiodine treatment in thyroid disorders.^36^ Indeed, ^131^I remains a mainstay in the treatment of autoimmune hyperthyroidism, toxic (multi-)nodular goiter, and differentiated thyroid cancer.^37,38^ Radioiodine treatment in thyroid disorders represented a blueprint for future “theranostics” approaches, combining the therapeutic scope of ^131^I treatment with the diagnostic value of pre- and post-therapeutic ^131^I scans.

In 2022, the US Food and Drug Administration and European Medicines Agency approved the use of [^177^Lu]Lu-PSMA-617, a ^177^Lu-labeled ligand of the prostate-specific membrane antigen (PSMA), for use in refractory, metastatic castration-resistant prostate cancer (mCRPC) after failure of anti-hormonal treatment and chemotherapy. The approval was based on the phase 3 VISION trial showing a significant progression-free (PFS) and overall survival (OS) benefit.^39^ More recent data confirmed the activity of [^177^Lu]Lu-PSMA-617 in taxane-naïve patients, showing a superiority in radiographic PFS compared to a change of androgen receptor pathway inhibitor as therapeutic strategy.^40^ The harnessed target PSMA is a transmembrane protein that is abundantly and almost exclusively expressed on both primary and metastatic prostate cancer cells, and predominantly in more aggressive tumors.^41,42^ This is underlined by the exceptional tumor-to-background ratio and high sensitivity of PSMA-PET, resulting in improved accuracy compared to conventional imaging by computed tomography and bone scan.^43^

Neuroendocrine tumors (NETs), especially those of gastroenteropancreatic origin, show strong expression of members of the SSTR family.^44–46^ Current guidelines recommend peptide receptor radionuclide therapy with [^177^Lu]Lu-DOTATATE based on the results of the NETTER-1 trial showing PFS superiority of [^177^Lu]Lu-DOTATATE compared to high-dose octreotide in patients with well-differentiated, SSTR-expressing midgut NETs after progression on monotherapy with long-acting octreotide.^47^ In this trial, [^177^Lu]Lu-DOTATATE treatment was well tolerated, and only 6% of patients treated with [^177^Lu]Lu-DOTATATE experienced serious treatment-related adverse events (grade ≥ 3) which mainly included hematological side effects and renal function impairment. Also in pancreatic NETs, [^177^Lu]Lu-DOTATATE was associated with higher 12-month PFS rates compared to sunitinib in the OCLURANDOM trial.^48^ Moving to earlier treatment lines, results of NETTER-2 revealed improved PFS of [^177^Lu]Lu-DOTATATE combined with low-dose octreotide compared to high-dose octreotide alone in newly diagnosed, SSTR-expressing, advanced grades 2 and 3 gastroenteropancreatic NETs.^49^ Of note, these are the first results in first-line treatment of [^177^Lu]Lu-DOTATATE in any solid tumor, highlighting the potential of this therapeutic strategy in earlier treatment lines.

## Early-Phase Data on Radionuclide Treatments in Brain Tumors

**Figure 1.**
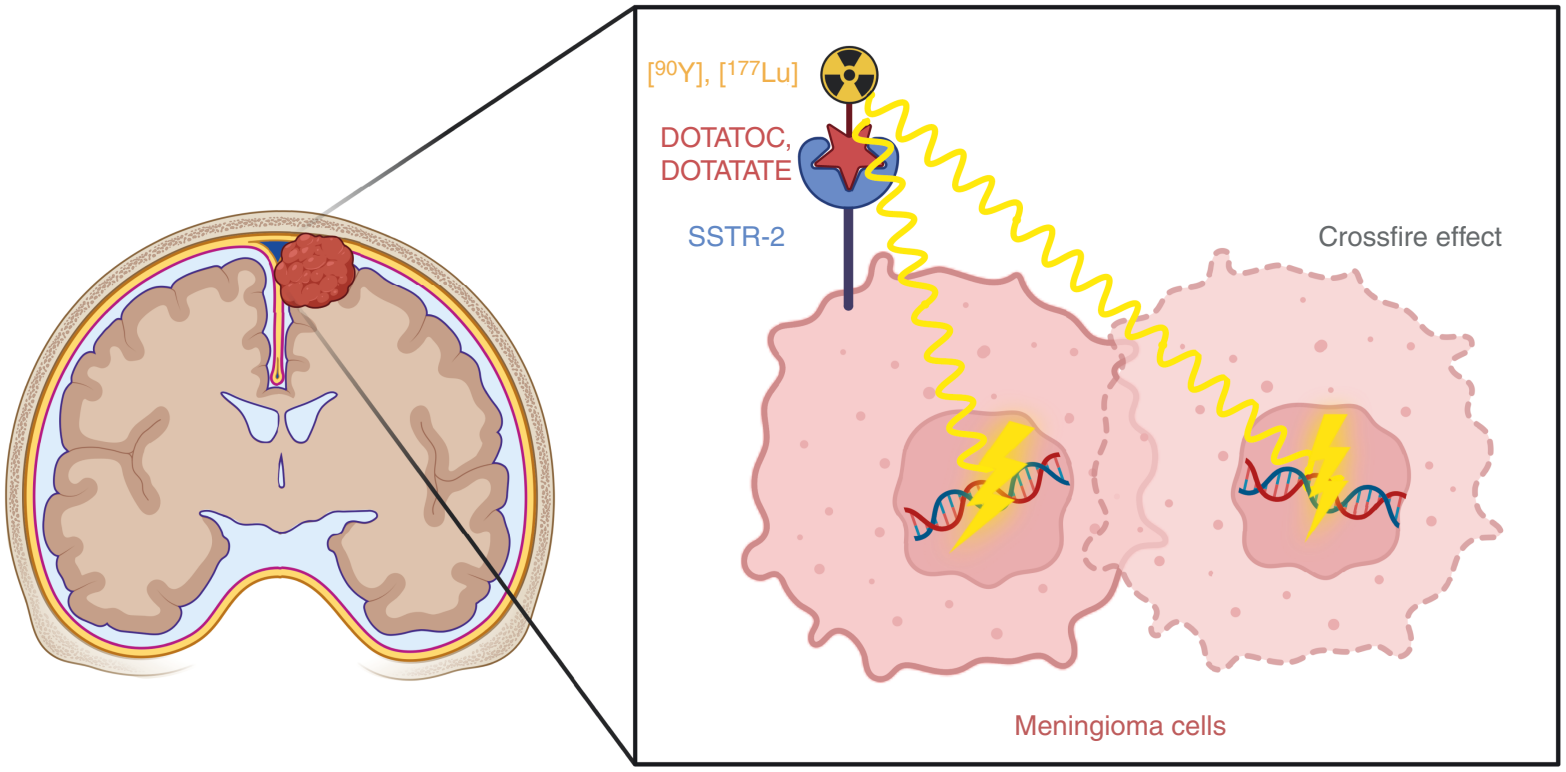
Overall concept of radioligand therapies in meningioma. DOTATATE, DOTA-Tyr3-octreotate; DOTATOC, DOTA-Tyr3-octreotide; SSTR-2, somatostatin receptor type 2. Figure created using BioRender.com.

Overall, treatment responses for CNS tumors are highly variable for targeted agents, probably due to intratumoral heterogeneity in target expression and the influence of the blood-brain/blood-tumor barrier (BBB/BTB). This also pertains to radionuclide treatments, especially if agents are injected intravenously.^50^ Similarly, antibody-drug conjugates, linking a cytotoxic drug to a tumor-specific antibody, show highly heterogeneous response rates across distinct entities of primary and secondary brain tumors, whereas the underlying biological mechanisms remain largely unclear.^51^ Here, the so-called “bystander” effect might considerably affect intracranial efficacy. Following binding of the antibody-drug conjugate to the target on the cell surface and endocytosis, the cytotoxic payload is released by proteolytic cleavage or due to the acidic milieu in cellular lysosomes. Depending on the lipophilicity, the payload can diffuse to adjacent cells and exert antineoplastic activity on tumor cells with lower target expression. Similarly, radionuclides enable neighboring cells to be targeted in a certain range depending on the emitter used, often referred to as “crossfire” effect (Figure 1).

Evidence for radionuclide treatments in brain tumors mainly stems from preclinical data and small case series. In glioma, targets such as epidermal growth factor receptor, l-type amino transporter 1 (LAT-1) neural cell adhesion molecule, glioma chloride channels, histone H1, neurokinin type 1 receptor, carbonic anhydrase XII, gastrin-releasing peptide receptor as well as the extracellular matrix protein tenascin-C have been investigated in small clinical trials as reviewed extensively elsewhere, with overall conflicting results.^52–55^ In addition, small case series also suggest therapeutic activity in (rarely occurring) brain metastases of prostate cancer treated with [^177^Lu]Lu-PSMA-617.^56^

### Meningioma

Evidence for the use of radionuclides in meningeal tumors is mainly based on small case series and early-phase prospective trials (Table 1). In most clinical trials of radioligand treatments in meningioma, patients were treated with [^177^Lu]Lu-DOTATATE as approved in NETs. In addition, other ^177^Lu-linked SSTR-2 ligands such as DOTATOC or ^90^Y-based radioligands were evaluated. Likewise, anecdotal data from multiple case reports are published where patients had been experimentally treated with SSTR-targeting approaches.^57–62^

**Table 1.** Clinical Data on Radionuclide Treatments in Meningioma

Compound	Type of study	Patient population	Number of included patients with meningioma	Outcome data (meningioma patients only)	Ref.
[^177^Lu]Lu-DOTATOC	Case series	Patients with paraganglioma, meningioma, small-cell lung cancer, and melanoma with positive uptake in [^111^In]-indium-pentetreotide scan after exhaustion of standard therapies	*n* = 5 -WHO grade III: 3 -Grade unknown: 2 (Other entities: 17)	PD in 3/5 (60%) patients, SD in 2/5 (40%) patients (according to routine CT/MRI)	van Essen et al. 2006^63^
[^90^Y]Y-DOTATOC	Prospective (no information on phase)	Patients with meningioma and positive SSTR-2 expression according to scintigraphy	*n* = 29 -WHO grade I: 14 -WHO grade II: 9 -WHO grade III: 6	SD in 19/29 (66%) patients, PD in 10/29 (34%). Median time to progression (TTP): 61 months in WHO grade I, 13 months in WHO grade II-III (according to routine MRI) Median OS: 69 months in WHO grade I, 30.5 months in WHO grade II-III	Bartolomei et al. 2009^64^
[^177^Lu]Lu-DOTATATE	Phase I-II	Patients with unresectable or metastatic SSTR-2-positive tumors (including NETs, paragangliomas, and meningiomas) by [^111^In]In-pentetreotide scintigraphy	*n* = 51 -Grade unknown: 1 (Other entities: 50)	No meningioma-specific outcome data provided	Bodei et al. 2011^65^
[^177^Lu]Lu-DOTATOC or [^177^Lu]Lu-DOTATATE followed by external beam radiotherapy	Prospective pilot trial (no information on phase)	Patients with unresectable advanced primary or recurrent meningioma	*n* = 10 -WHO grade I: 7 -WHO grade II: 2 -Grade unknown: 1	CR in 1/10 (10%) patients, PR in 1/10 (10%) patients, SD in 8/10 (80%) patients according to routine MRI PET-positive volume reduction to median of 81% of pretreatment size; SUVmax increase by a median of 37% (7 evaluable patients) Long-term outcome data: CR in 1/10 (10%), SD in 6/10 (60%), PD in 3/10 (30%); median PFS 91.1 months; median OS 105 months.	Kreissl et al. 2012^66^ (long-term outcome data in Hartrampf et al. 2020^67^)
[^177^Lu]Lu-DOTATOC or [^177^Lu]Lu-DOTATATE	Prospective pilot trial (no information on phase)	Patients with primary meningioma of WHO grade 2 and 3 or recurrent meningioma of any grade or meningioma ≥ 5 cm diameter with adjacent critical structures and positive SSTR-2 PET	*n* = 11 (no information on individual WHO grades)	Correlations between (1) maximum voxel dose and activity retention 1 hour after administration of [^177^Lu]Lu-DOTATOC or [^177^Lu]Lu-DOTATATE and (2) SUVmax in pre-therapeutic PET; no information on treatment response	Hänscheid et al. 2012^68^
[^111^In]In-pentetreotide (in some patients combined with [^90^Y]Y-DOTATOC and [^90^Y]Y-DOTATATE)	Retrospective case series	Patients with meningioma or meningiomatosis	*n* = 8 -WHO grade I: 5 -WHO grade II: 3	PR in 2/8 (25%) patients, SD in 5/8 (62.5% patients), PD in 1/8 (12.5%) case	Minutoli et al. 2014^69^
[^90^Y]Y-DOTATOC	Phase II	Patients with SSTR-2a-positive (by [^111^In]In-DOTATOC or [^111^In]In-pentetreotide scintigraphy), recurrent or progressive meningiomas in functionally critical areas or not amenable to surgery due to unfavorable medical risk profile or refusal of surgery	*n* = 15 -WHO grade I: 9 -WHO grade II: 2 -WHO grade III: 1 -Unknown: 3	SD in 13/15 (87%), PD in 2/15 (13%) of patients Median PFS: 24 months	Gerster-Gilliéron et al. 2015^70^
[^177^Lu]Lu-DOTATOC or [^90^Y]Y-DOTATOC	Phase II	Patients with progressing meningioma and uptake on SSTR-2 scintigraphy	*n* = 37 -WHO grade I: 5 -WHO grade II: 6 -WHO grade III: 3 -Grade unknown: 23	SD in 23/34 (68%), PD in 11/34 (32%) Mean OS: 8.6 years	Marincek et al. 2015^71^
[^177^Lu]Lu-DOTATATE or [^90^Y]Y-DOTATOC, or both	Retrospective case series	Patients with progressing meningioma and positive SSTR-2 PET or scintigraphy	*n* = 20 -WHO grade I: 5 -WHO grade II: 7 -WHO grade III: 8 (WHO grades for surgery at recurrence)	WHO grade I: -SD in 5/5 (100%) -Median PFS: 32.2 months -Median OS: not reached WHO grade II: -SD in 4/7 (57%), PD in 3/7 (43%) -Median PFS: 7.6 months -Median OS: not reached WHO grade III: -SD in 1/8 (12.5%), PD in 7/8 (87.5%) -Median PFS: 2.1 months -Median OS: 17.2 months	Seystahl et al. 2016^72^
[^177^Lu]Lu-DOTATATE	Retrospective case series	Patients with advanced/metastatic NET and incidentally diagnosed [^68^Ga]Ga-DOTATATE-positive lesions with high suspicion of meningioma based on MRI	*n* = 5 (WHO grades unknown)	CR in 2/5 (40%), PR in 1/5 (20%), PD in 2/5 (40%). Median PFS (meningioma-related): 26 months.	Parghane et al. 2019^73^
[^177^Lu]Lu-DOTATATE	Retrospective case series	Patients with progressive intracranial meningioma	*n* = 7 -WHO grade I: 2 -WHO grade II: 5	SD in 2/4 (50%) evaluable patients (according to PET) PFS at 6 months: 42.9%	Müther et al. 2020^74^
[^177^Lu]Lu-DOTATOC	Retrospective case series	Patients with confirmed neurofibromatosis type 2 and multiple partially pretreated meningiomas and SSTR-2 expression by [^68^Ga]Ga-DOTATOC	*n* = 11 -WHO grade I: 4 -WHO grade II: 6 -WHO grade III: 1	SD in 6/11 (55%), PD in 5/11 (45%) Median PFS: 4 months Median OS: 50 months	Kertels et al. 2021^75^
[^177^Lu]Lu-DOTATATE	Retrospective case series	Patients with progressive meningioma and no further surgical or radiotherapeutic treatment options and SSTR-positive lesions in PET	*n* = 8 -CNS WHO grade 2: 8	SD in 7/8 (87.5%), PD in 1/8 (12.5%) - according to RANO criteria on MRI PFS at 6 months: 85.7%	Salgues et al. 2022^76^
[^177^Lu]Lu-DOTATATE	Retrospective case series	Patients with progressive meningioma not amenable to further surgery or radiotherapy	*n* = 15 -WHO grade I: 3 -WHO grade II: 5 -WHO grade III: 6 -Grade unknown: 1	SD in 6/15 (40%), PD in 8/15 (53%), one death during treatment—according to RANO criteria on MRI Median PFS: 7.8 months	Minczeles et al. 2023^77^
[^177^Lu]Lu-DOTATATE	Phase II	Patients with progressive meningioma of all grades	*n* = 14 on interim report (32 planned) -WHO grade 1: 2 -WHO grade 2: 11 -WHO grade 3: 1	SD in 9/14 (64%), PD = 4/14 (29%), lost-to-follow-up in 1 patient PFS at 6 months: 50% Median PFS = 8.2 months Median OS = 21.9 months >25% SUV reduction in [68Ga]Ga-DOTATATE in 5 lesions	Kurz et al. 2024^78^

CR, complete remission; CT, computed tomography; DOTATATE, DOTA-Tyr3-octreotate; DOTATOC, DOTA-Tyr3-octreotide; MRI, magnetic resonance imaging; NET, neuroendocrine tumor; OS, overall survival; PD, progressive disease; PET, positron emission tomography; PFS, progression-free survival; PR, partial remission; RANO, response assessment in neuro-oncology; SD, stable disease; SSTR-2, somatostatin receptor type 2; SUV_max_, maximum standardized uptake value; TTP, time to progression; WHO, World Health Organization.

In an early-phase prospective trial in 29 patients with meningioma of all grades, Bartolomei et al. observed that treatment with [^90^Y]Y-DOTATOC resulted in stable disease (SD) in 66% of included patients, while progressive disease was seen in 34%. Of note, [^90^Y]Y-DOTATOC treatment did not result in shrinkage of lesions, and stabilization of lesions was more frequently observed in WHO grade 1 meningioma than in higher-grade lesions.^64^ Similar results were seen in other studies, and complete (CR) or partial responses were overall rarely reported regardless of the compound used.^66,69,73^ In line with these findings, the largest trial to date in meningiomas of all grades confirmed uptake in SSTR-2 scintigraphy (*n* = 37) and showed SD in 68% and progressive disease in 32% of patients treated with [^177^Lu]Lu-DOTATOC or [^90^Y]Y-DOTATOC.^71^ A retrospective case series of patients treated with [^177^Lu]Lu-DOTATATE and/or [^90^Y]Y-DOTATOC showed SD in 100% of WHO grade 1 meningioma, whereas WHO grade 2 and 3 lesions had SD in 57% and 12.5%, respectively.^72^ These results were corroborated by 3D volumetry. Analysis of maximum and mean standardized uptake values (SUV_max_, SUV_mean_) in SSTR PET, as well as immunohistochemical SSTR-2 staining, showed better responses in meningiomas with higher SSTR-2 expression, underscoring the predictive value of target expression assessed by both molecular imaging and in tumor tissue. In another recent publication, ≥25% reduction in SUV was observed in 5/13 measurable lesions upon [^177^Lu]Lu-DOTATATE treatment, which correlated with disease control and therefore highlights the potential of response monitoring by molecular imaging.^78^

Data on the toxicity of [^177^Lu]Lu-DOTATATE treatment in meningioma are scarce.^72,76^ In one retrospective publication, lymphocytopenia was most frequently observed (70% of patients), where lymphocyte counts correlated with the number of previous systemic treatment lines. Other observed side effects included anemia, thrombocytopenia, increase of gamma-glutamyltransferase, fatigue, alopecia, pituitary insufficiency, and wound complications.^72^

Despite these studies, high-grade evidence for the use of radionuclide therapies in meningioma is lacking. Some studies included SSTR-positive tumors regardless of the underlying entity, including also NETs, paragangliomas, small-cell lung cancer, and melanoma, and meningioma-specific outcome measures were not reported.^63,65^ While some publications suggest that the antitumoral activity might be more pronounced in more benign and lower-grade tumors, data on WHO grades is frequently missing. Furthermore, tumor grading and classification have undergone considerable changes in the past decades, limiting the applicability of these results to the integrated molecular diagnostic framework currently in use.^2^ Additionally, response assessment was not uniformly conducted across published data, and older reports use arbitrary cutoffs to define treatment response or adapted MacDonald criteria for response assessment in malignant glioma. Only recently, standardized criteria for MRI-based response assessment in meningioma were proposed by the RANO working group,^79^ and currently, there is no generally accepted framework for the standardized assessment of SSTR-based PET imaging in meningioma. In addition, meningiomas frequently show slow growth rates, complicating response assessment given the short follow-up in many publications. Here, 3-dimensional volume growth rate (3DVGR) measurements might be helpful as observed in a recent post hoc analysis of EORTC-1320, a prospective trial in higher-grade meningioma.^80^ These were shown to correlate with 6-month PFS and may provide a more refined response classification compared to modified Macdonald criteria.

## Future Outlooks

### Choice of Radionuclide Emitter

To date, data on radionuclide treatment in meningioma are almost exclusively based on radioligands with the β^-^ emitters ^177^Lu or ^90^Y. However, their linear energy transfer is relatively low compared to ɑ particle emitters such as ^225^Ac or ^223^Ra (Table 2). In specific, β^-^ particles predominantly act by forming reactive oxygen species (ROS) which lead to single-strand breaks, whereas ɑ particles elicit double-strand breaks by direct interaction with DNA, causing higher lethality due to limited DNA repair mechanisms. Indeed, there are no known biological intrinsic resistance mechanisms for ɑ particles so far.^81^ In addition, the limited range and therefore reduced crossfire effect allows sparing of adjacent tissues while specifically targeting high radiation doses to the tumor, which is of particular significance in brain tumors. This is also underscored by the importance of target delineation in external beam radiotherapy of meningiomas as outlined in recent consensus guidelines.^82^ On the other hand, the short range of ɑ particles might confer less activity in heterogeneous tumors where some cells show lower target expression. Mixed ɑ/β treatments might therefore combine the higher linear energy transfer of ɑ particles with the increased crossfire effect of β particles, but further studies on efficacy and safety are needed.

**Table 2.** Overview of Particle-Specific Properties of Therapeutic Radionuclides

	ɑ particles	β^−^ particles	Auger electrons
Isotopes	[^225^Ac], [^223^Ra], [^212^Pb]	[^131^I], [^90^Y], [^177^Lu]	[^125^I], [111In]
Range	10–100 µm	1–10 mm	~0.1 µm
Linear energy transfer	50–300 keV/µm	0.2 keV/µm	4-26 keV/µm
DNA damage	DNA double-strand breaks by direct interaction with DNA	DNA single-strand breaks by reactive oxygen species	Direct and indirect damage to DNA (single-/double-strand breaks), reactive oxygen species, damage of phospholipid bilayer in membranes

Use of the ɑ emitter ^223^Ra is currently approved in bone-metastatic, mCRPC based on the phase 3 ALSYMPCA trial.^83^ Similar to the application of iodine isotopes in thyroid disorders, ^223^Ra is delivered to the target tissue by mimicking calcium atoms which are absorbed in metastatic bone tissue remodeling. Recently, results of a large retrospective cohort of mCRPC patients treated with the ɑ emitter-based radioligand [^225^Ac]Ac-PSMA were published, showing antitumoral activity at an acceptable side effect profile.^84^ In NETs previously treated with ^177^Lu-based agents, [^225^Ac]Ac-DOTATATE showed promising results in early pilot studies,^85,86^ but data in meningioma are lacking so far.

Auger electrons have an even lower range than ɑ particles, thereby reducing toxicity to neighboring tissues. Physically, radioactive decay of nuclides leads to transitions in the electron shell of atoms, releasing energy in the form of X-rays or Auger electrons. These cause direct damage to DNA by eliciting double-strand breaks, but also indirect cell damage through the formation of reactive oxygen species and the interaction with the phospholipid bilayer of the cell membrane have been reported.^87^ While research efforts in the past years mainly focused on ɑ therapies, the clinical activity and safety of Auger electron therapeutics are not well established. Indeed, most data on Auger electron treatment in cancer is based on [^111^In]In-pentetreotide treatment in NETs, with overall promising treatment responses and mainly transient, mild hematotoxicity.^88–90^ In meningioma, only one small case series of 8 patients receiving [^111^In]In-pentetreotide with or without [^90^Y]Y-DOTATOC has been published so far, and larger prospective trials are needed to substantiate early signs of antitumoral activity.^69^

More recently, results of a dosimetric study with ^64^Cu/^67^Cu-linked to octreotate by a sarcophagine MeCOSar chelator ([^64^Cu/^67^Cu]Cu-SARTATE) in patients with unresectable meningiomas have been reported. The β-emitting isotope pair ^64^Cu/^67^Cu has reduced half-lives compared to ^177^Lu, allowing for more frequent administration and easier logistics in terms of radiation protection regulations. Moreover, the positron-emitting ^64^Cu used for diagnostic imaging may more accurately predict the biodistribution of the therapeutic ^67^Cu than in theranostic pairs based on different elements such as ^68^Ga and ^90^Y or ^117^Lu. While tolerability was acceptable, efficacy remains to be demonstrated.^91^

### Improving Dosimetry

Classical pharmacotherapy follows either fixed-dose regimens or is based on body weight or body surface according to pharmacokinetic data. In radionuclide treatment, absorbed doses can vary considerably between different individuals, affecting both therapeutic responses in the target tissue as well as potential side effects in organs at risk, mainly kidneys and bone marrow. While effective and maximum tolerated dose thresholds are standardized in conventional radiotherapy, dose–response, and dose-toxicity relationships are less clearly defined in radionuclide therapies.^92^ Indeed, dose thresholds are frequently extrapolated from external beam radiotherapy, and fixed-dose regimens based on conventional dose escalation trials are currently in use for all approved treatments including [^177^Lu]Lu-DOTATATE, [^177^Lu]Lu-PSMA, and ^223^Ra. For instance, the approved dosing regimen of [^177^Lu]Lu-DOTATATE in NETs includes 4 courses with 7400 MBq every 8 weeks. Other schedules are being explored, also in brain tumors such as glioblastoma where administration every 4 weeks is being evaluated in a dose-finding study of [^177^Lu]Lu-DOTATATE (NCT05109728).

Dosimetry enables the collection of further information on absorbed radiation doses in the target tissue and other organs that may be affected by adverse events. As a basic requirement, patient-averaged dosimetry in standardized nuclear medicine treatments is mandatory according to current regulations in the European Union.^93^ However, this does not extend to organ- or tissue-specific absorbed radiation doses which would open new avenues to introduce personalized dosing schemes to radionuclide treatments. In specific, future approaches could allow to improve on-target efficacy and spare off-target toxicity by systematically collecting data on tissue-specific absorbed radiation doses.

Radionuclides emitting ɣ rays such as ^177^Lu allow collection of planar as well as 3D SPECT images to monitor the distribution of radioactive activity. In contrast, ^90^Y does not emit ɣ radiation. While other radionuclides such as ^111^In may be employed as surrogate radionuclides for dosimetry, ^111^In dosimetry does not correlate well with the biological effects of ^90^Y-based treatment.^94^ In addition, dosimetry protocols are frequently not harmonized between institutions, challenging the overall acceptance in the nuclear medicine community.^95^ While results of clinical trials indicating a superiority of personalized radionuclide treatment are so far missing, early results in NETs are promising. Indeed, the cumulative maximum tumor dose could be increased by the factor 1.26 in a prospective trial, whereas the occurrence of side effects was similar to fixed-dose regimens.^96^

In meningioma, dosimetry information has rarely been reported in published data on radionuclide treatment. In the recently published retrospective analysis by Minczeles et al., absorbed doses were highly heterogeneous and ranged from 7 to 404 Gy in 14 target tumors of 8 patients.^77^ Another trial aimed to predict dosimetric data based on pre-therapeutic [^68^Ga]Ga-DOTATOC PET imaging in meningioma. Here, correlations between SUV_mean_-derived values in [^68^Ga]Ga-DOTATOC PET performed prior to [^177^Lu]Lu-DOTATATE and tumor-absorbed doses were observed^97^. However, the high heterogeneity of applied doses as well as the paucity of data underscore the urgent need for systematic collection of data on dosimetry in radionuclide treatment trials of brain tumors and meningiomas.

### Route of Administration

Currently approved radionuclide treatments such as [^177^Lu]Lu-DOTATATE, [^177^Lu]Lu-PSMA, and ^223^Ra are generally administered intravenously. In general, systemic administration is complicated by the BBB/BTB, although meningiomas are usually located outside these barriers. In intraaxial tumors, multiple strategies to disrupt and bypass the BBB/BTB have been described, including cellular, molecular, and physical/chemical approaches as extensively reviewed elsewhere.^98^

For radionuclide treatments, studies on intraventricular and intracavitary applications have been performed. For instance, intraventricular application of [^131^I]I-omburtamab, an antibody targeting the tumor antigen B7-H3 conjugated to ^131^I, has been evaluated in recurrent medulloblastoma and ependymoma in a phase 1 trial, with an acceptable side effect profile and signs of antitumoral activity as compared to historical data.^99^ In glioma, intracavitary administration of a conjugate consisting of ^131^I and the monoclonal anti-tenascin antibody 81C6 ([^131^I]I-81C6) has shown an acceptable side effect profile and compared favorably with historical controls, but further development was discontinued.^100^ Similar results were seen with [^211^At]At-81C6.^101^ Moreover, radionuclide treatment with a nano-liposomal formulation of ^186^Rh has been explored using pressure gradients through intratumoral and intracavitary application in glioma (also known as convection-enhanced delivery). While overall results were promising, the absorbed doses were highly heterogeneous and future studies are needed to substantiate an improvement in treatment outcomes compared to external beam radiotherapy.^102^

In meningioma, intraarterial delivery may represent a promising concept to maximize intratumoral doses and minimize systemic toxicity. The feasibility of this approach has initially been demonstrated in a diagnostic [^68^Ga]Ga-DOTATATE PET study, where intraarterial application of the tracer increased SUV_mean_ values by 2.7-fold compared to systemic intravenous administration.^103^ One case report of [^177^Lu]Lu-DOTATATE treatment in meningioma showed improved tumor uptake compared to intravenous radionuclide treatment.^60^ Another investigation in 4 patients with treatment-refractory meningiomas confirmed these findings, and no significant adverse effects were observed.^104^ Further clinical research is warranted, especially as intraarterial administration showed conflicting results in other tumors such as NETs metastatic to the liver.^105,106^

### Optimal Clinical Trial Design

Clinical trials in rare and heterogeneous entities such as treatment-refractory meningioma remain challenging. Similar to systemic treatment in meningioma, the evidence for radionuclide therapy is based on retrospective data or small prospective pilot trials, and most included patients are heavily pretreated with no further treatment options.^9^ Indeed, controlled trials comparing radioligand therapy to other therapeutic approaches in a randomized fashion are lacking, but ultimately needed to define efficacy and safety. This also includes well-designed clinical trials of radionuclide treatment strategies in earlier treatment lines. Moreover, response assessment in available data varies considerably, challenging the interpretation of available outcome data and the use of external controls which is increasingly considered in rare oncological entities where the implementation of randomized controlled trials is not always feasible.^107^

The European Organization for Research and Treatment of Cancer—Brain Tumor Group (EORTC-BTG) has recently formulated considerations for clinical trial design and conduct in the field of radionuclide treatment.^108^ These include the endorsement for controlled trials with well-defined endpoints such as OS and PFS based on consensus recommendations for response assessment such as those of the RANO consortium.^79^ Moreover, the standardized collection of patient-reported outcomes is crucial as intracranial tumors including meningiomas considerably impact physical, neurocognitive, emotional, and social functioning.^109,110^ From a technical point of view, the systematic acquisition of dosimetric data would allow for improvement in dosing schedules, including optimized length of cycles as well as intratumoral absorbed doses while minimizing systemic toxicity. In addition, harmonization of regulatory frameworks and institutional protocols is a prime prerequisite for the conduct of clinical trials and implementation of standardized treatment schemes in clinical routine.^25^

In addition, translational research efforts within clinical trials are needed to detect and validate predictive biomarkers. Among these lines, recent data have shown that targeted gene expression profiling allows to identify cases who benefit from postoperative radiotherapy.^7^ Involved genes include members of pathways concerned with cell cycle and mitotic stability, suggesting that these alterations might also confer higher sensitivity to other cytotoxic treatments such as radionuclide therapies. However, further validation in prospective trials is necessary.

## Ongoing Clinical Trial Initiatives

Currently, ongoing clinical trials are given in Table 3. The vast majority of trials aim to investigate [^177^Lu]Lu-DOTATATE and [^177^Lu]Lu-DOTATOC in meningiomas of different grades. ELUMEN (NCT06126588) aims to evaluate the combination of [^177^Lu]Lu-DOTATATE with everolimus based on promising data on systemic treatment of combined mammalian target of rapamycin (mTOR) and SSTR-2 inhibition.^111^ The crossover phase 0/I/II trial PROMENADE (NCT04997317) aims to compare [^177^Lu]Lu-DOTATOC with [^177^Lu]Lu-satoreotide in a 2-step trial design. Indeed, [^177^Lu]Lu-satoreotide has shown higher intratumoral absorbed doses compared to [^177^Lu]Lu-DOTATATE, probably given the improved binding of the SSTR antagonist satoreotide compared to the agonist DOTATATE.^112^ First results of the phase 0 arm of PROMENADE have been published recently,^113^ with higher tumor-to-bone marrow and tumor-to-kidney ratios in patients receiving [^177^Lu]Lu-satoreotide compared to [^177^Lu]Lu-DOTATATE. In addition, early signs of efficacy have been observed as the disease control rate was 83%. Further investigation in the phase I/II arm is ongoing.

**Table 3.** Overview of Ongoing Clinical Trials on Radionuclide Treatments in Meningioma

Clinical trial identification/trial name	Phase	Study design	Compound	Main inclusion criterion	Status (effective January 29, 2024)
NCT06126588 (ELUMEN)	Phase II	Single-arm	[^177^Lu]Lu−DOTATATE + everolimus	Meningioma WHO grades 2 and 3 (histologically verified) with progression and not amenable to surgery or RT and positive in [^68^Ga]Ga-DOTATOC PET	Not yet recruiting
NCT04997317 (PROMENADE)	Phase 0/I/II	Two-step, cross-over open-label phase 0 followed by single-arm open-label phase I/II	[^177^Lu]Lu−DOTATOC, [^177^Lu]Lu−DOTA-JR11 ([^177^Lu]Lu−satoreotide)	Recurrent or progressive meningioma (either histologically confirmed or high suspicion based on MRI and SSTR imaging)	Recruiting
NCT03971461	Phase II	Single-arm	[^177^Lu]Lu−DOTATATE	Meningioma WHO grade 1 (progressing after surgery and RT or progressive residual tumor after surgery not amenable to RT) or meningioma WHO grades 2 and 3 (progressing after surgery and RT or residual measurable disease after surgery)	Recruiting
NCT04082520	Phase II	Single-arm	[^177^Lu]Lu−DOTATATE	Progressive meningioma with measurable disease after previous surgery and RT (not amenable to further RT)	Recruiting
NCT03273712	Phase II	Single-arm	[^90^Y]Y−DOTATOC	Pediatric and adult patients with SSTR-2-positive tumors according to [^68^Ga]Ga-DOTATOC/DOTATE PET (including NET, meningioma, neuroblastoma, and medulloblastoma) not amenable to standard treatment after failure of first-line treatment	Completed (no results published)
NCT05278208	Phase I/II	Two-step, single-arm phase I/II	[^177^Lu]Lu−DOTATATE	Pediatric and young adult patients with recurrent/progressive high-grade CNS tumors and meningiomas expressing SSTR-2A and showing uptake in DOTATATE PET	Recruiting

DOTATATE, DOTA-Tyr3-octreotate; DOTATOC, DOTA-Tyr3-octreotide; MRI, magnetic resonance imaging; NET, neuroendocrine tumor; PET, positron emission tomography; RT, radiotherapy; SSTR-2, somatostatin receptor type 2; WHO, World Health Organization.

Within EORTC-BTG-2334 (LUMEN-1), intravenous [^177^Lu]Lu-DOTATATE will be assessed in recurrent or progressing, SSTR-2-expressing meningiomas of all grades after surgery and radiotherapy. For the first time in meningioma, radionuclide treatment will be compared to local standard of care in a randomized fashion with PFS as primary endpoint, and OS, tolerability, and quality of life as secondary endpoints. Within this trial, translational and further patient-reported outcome data will be systematically collected to define efficacy, safety and potential predictive markers impacting therapeutic responses. Of special interest is the planned collection of dosimetry data as this will allow us to study the inter-individual variability of absorbed doses and their correlation with treatment response.

## Conclusion

In conclusion, radionuclide therapies represent a promising treatment approach in meningioma. Data from small clinical trials and retrospective case series demonstrate safety and suggest therapeutic activity in progressing tumors after failure of local treatment, but evidence is still limited. In addition, the utility of these agents earlier in the treatment course of meningioma remains to be established. Further innovative strategies in drug design, such as changes in the employed radionuclide or the used ligand, are being explored in ongoing studies. Alternative dosing regimens (such as shorter application intervals) and combination therapies, for instance with radiosensitizers or immunotherapies, may be explored in order to induce synergistic efficacy. Furthermore, alternative administration routes including intraarterial administration as well as further collection of dosimetry data may lead to improved intratumoral doses and reduced systemic exposure, resulting in even higher therapeutic responses and decreased off-target toxicity. Overall, well-designed trials are urgently needed to define the value of radioligand therapies and related novel approaches in meningioma.
